# *Anabasis setifera* leaf extract from arid habitat: A treasure trove of bioactive phytochemicals with potent antimicrobial, anticancer, and antioxidant properties

**DOI:** 10.1371/journal.pone.0310298

**Published:** 2024-10-25

**Authors:** Amer M. Abdelaziz, Mostafa A. Abdel-Maksoud, Sabiha Fatima, Saeedah Almutairi, Bushra Hafeez Kiani, Amr H. Hashem

**Affiliations:** 1 Faculty of Science, Botany and Microbiology Department, Al-Azhar University, Cairo, Egypt; 2 Botany and Microbiology Department, College of Science, King Saud University, Riyadh, Saudi Arabia; 3 Department of Clinical Laboratory Science, College of Applied Medical Sciences, King Saud University, Riyadh, Saudi Arabia; 4 Department of Biology and Biotechnology, Worcester Polytechnic Institute, Worcester, Massachuesetts, United States of America; South Valley University Faculty of Agriculture, EGYPT

## Abstract

The main objective of this study was to evaluate the biological activities of *Anabasis setifera* extract, including its antimicrobial, anticancer, and antioxidant properties. In the current study, *Anabasis setifera* leaves extract was evaluated for antimicrobial, anticancer, antioxidant activities and phytochemical analyses. Ethyl acetate extract of *Anabasis setifera* (EA-AS) exhibited promising antimicrobial activity toward *Escherichia coli*, *Staphylococcus aureus*, *Salmonella typhimurium*, *Bacillus subtilis*, *Candida albicans*, *Aspergillus brasiliensis*, *Aspergillus fumigatus* with MICs 62.5, 125, 62.5, 31.25, 62.5, 125 and 125 μg/mL respectively. Moreover, EA-AS showed anticancer activity at safe concentrations, where IC_50_ were 36.4 and 44 μg/mL toward Hep-G2 and MCF-7 cancerous cell lines. EA-AS was found to contain 55 significant compounds identified through gas chromatography mass spectrophotometry (GCMS). The most abundant compounds were 1,4-dimethoxy-6,7,8,9-tetrahydro-5-benzocycloheptenone (26.04%), hexa-2,4-diyn-1-ylbenzene (8.40%), dihydrobenzo[b]fluoranthene (6.10%), ethanone, 1-[2,3-dihydro-2-(1-methylethenyl)-5-benzofuranyl (6.10%), and valerenol (4.08%). GC mass analysis confirmed the antioxidant properties of AS by detecting several compounds with antioxidant activity, including hexa-2,4-diyn-1-ylbenzene, nerolidol, spathulenol, -naphthalenem ethanol, decahydro-4-trimethyl-8-methylene, hexadecenoic acid, tremetone, desmethoxyencecalin, heptadecyn-1-ol, thunbergol, hexadecanol, dotriacontane, taylorione, ligulatin, retinoic acid, and falcarinol. The analysis of EA-AS reveals that it is a rich source of valuable phytochemicals: total Phenolic Content: a promising 4,264 μg/mL /, suggesting substantial biological and pharmacological potential. Total tannin content: 391.17 μg/mL, indicating potential applications in industries like nutraceuticals, pharmaceuticals, and cosmetics. Total flavonoid content exceptionally high at 5,163 μg/mL, while the total alkaloid content measured 1,036.26 μg/mL. Additionally, EA-AS demonstrated antioxidant activity with an EC_50_ of 30.6 μg/mL. In conclusion, the comprehensive analysis of the EA-AS reveals its immense potential as a rich source of valuable phytochemicals with diverse bioactivities, warranting further in-depth studies to unlock its full pharmaceutical and commercial prospects. Our results suggest substantial biological and pharmacological prospects for EA-AS as a promising antimicrobial, anticancer, and potent antioxidant.

## 1. Introduction

Antimicrobial resistance (AMR) is a serious and increasing global health problem that occurs when microbes, including bacteria, viruses, and fungi, gain the capability to resist the effects of antimicrobial medications [1,2]. As a result, common treatments become ineffective, leading to prolonged illnesses, increased hospitalizations, and higher mortality rates [3]. AMR is particularly concerning in the context of cancer care, as cancer patients often have weakened immune systems that make them more susceptible to infections [1,4]. The rise of AMR is jeopardizing the progress made in cancer treatment, as oncologists are finding that their go-to antibiotics and antifungal medications are no longer effective against the infections that commonly afflict cancer patients [5]. Addressing AMR through improved antibiotic stewardship and the development of new antimicrobial agents is crucial for ensuring that cancer patients can receive the full course of their essential cancer treatments and have access to effective antimicrobial agents [6].

Cancer remains one of the leading causes of death worldwide, with many cancer types still lacking effective treatments. Traditional cancer drugs often have limited efficacy, with many patients either not responding or eventually developing resistance to the medications [7,8]. This can lead to disease progression and poor outcomes for patients. Additionally, these traditional therapies frequently come with significant side effects that can negatively impact a patient’s quality of life during treatment [8]. While progress has been made in developing more targeted and personalized cancer therapies, there remains an urgent need for continued research and development of new, more effective treatment strategies that can improve patient outcomes and quality of life [7,9]. This challenge highlights the importance of having alternatives to currently used materials that are more efficient and safer for humans and the environment. Therefore, a natural and safe source was considered for testing to address these challenges.

*Anabasis setifera* (AS) is a plant that belongs to the kingdom Plantae. It is classified within the phylum Tracheophyta, class Magnoliopsida, order Caryophyllales, Amaranthaceae family, under the subfamily Salsoloideae and the tribe Salsoleae. AS is a hardy, perennial plant that excels in extremely arid environments. This resilient species has developed unique adaptations to survive in areas characterized by high salinity and frequent droughts [10]. AS is a compact, woody subshrub that can grow up to 50 cm tall. It has a dense, branching stem covered in greyish-green, scale-like leaves that are succulent and help conserve water. The leaves are covered in trichomes, giving the plant a bristly appearance. The small, greenish-white flowers are arranged in terminal spikes or clusters and are not particularly noticeable. The plant produces small, one-seeded fruits called utricles that are designed for wind or animal dispersal. The genus *Anabasis* is known to be a rich source of biologically active secondary compounds (BASC), such as sesquiterpenes, diterpenes, triterpenes, saponins, phenolic acids, flavonoids, and betalain pigments. These diverse phytochemicals contribute to the wide range of pharmacological activities exhibited by *Anabasis* species [11]. The promising properties of AS suggest its potential as an effective, safe, and non-traditional treatment to limit the spread of pathogens resistant to conventional treatments. AS is found in various habitats in Egypt, including all Egyptian deserts and coastal areas due to the unique characteristics of AS highlight the plant’s adaptability to various environments and its ability to thrive in diverse ecological conditions [12]. The current study aimed to comprehensively evaluate the biological activities of the ethyl acetate extract derived from the leaves of the plant *Anabasis setifera* (EA-AS). This assessment will be coupled with a detailed phytochemical analysis of the extract to identify the key bioactive compounds responsible for these activities. The results of this investigation contribute to the growing body of knowledge on the therapeutic potential of AS and its possible applications in various industries, including medicine, pharmaceuticals, and cosmetics.

## 2. Materials and methods

### Plant material

AS leaf samples were collected from Ain-Sokhna-Alqattamiya road, Suez, Egypt. The plant was identified by Prof. Dr. Abdou Marie Hamed from the Botany and Microbiology Department, Faculty of Science, Al-Azhar University, Cairo, Egypt. The plant was kept in Faculty of Science herbarium, Al-Azhar University (Voucher no. 775). The experimental research and field studies, including the collection of plant material, were conducted in compliance with the relevant institutional, national, and international guidelines and legislation.

### Extraction of bioactive metabolites

To prepare the AS extracts, 10 grams of powdered leaf materials were extracted using 100 ml of ethyl acetate. The solvent layer was separated using a separating funnel and then evaporated. AS extract was used as a stock for subsequent experiments.

### Antimicrobial activity

The preliminary qualitative antibacterial effect of EA-AS extract was assessed following the agar well diffusion assay. The activity was evaluated against Gram-positive *B*. *subtilis* ATCC 6051 and *S*. *aureus* ATCC 25923 and Gram-negative *S*. *typhimurium* ATCC 14028 and *E*. *coli* ATCC 25922. All steps were performed under aseptic condition according to the Clinical Laboratory Standard Institute (CLSI) guiding principle [13]. Briefly, each tested bacterial strain, equivalent to 0.5 McFarland standard concentrations, was inoculated with sterile cotton swab on the surface of Mueller-Hinton agar (MHA) palate. Using sterile cork-borer, wells of 8 mm was made on the agar surface and 100 μL of the tested EA-AS extract (1000 μg/ml)/ethyl acetate (EA)/ SAM (1000 μg/ml)/ Fluconazole (1000 μg/ml) was separately placed into the obtained wells. After incubation at 37°C for 24 h for bacteria and 30°C for 48 and 96 h for fungi, each inhibition zone diameter was measured.

Additionally, the quantitative antibacterial activity of the EA-AS extract towards the tested stains was determined via broth microdilution assay. In brief, the crude extract was suspended in DMSO and tryptic soy broth (TSB) for preparation of stock concentration (1000 μg/ml) and two-fold serial dilution was created in 96-well microtiter plates. Each bacterium inoculum (10 μl) equivalent to 0.5 McFarland standard was then inoculated. Positive control containing the tested bacterial isolate in TSB and DMSO without the crude extract was included while, the applied negative control was consisted of TSB, DMSO, and crude extract without bacterial inoculum. After incubation at 37°C for 24 h, the lowest EA-AS concentration associated with no bacterial growth was recorded as the minimum inhibitory concentration (MIC).

### Cytotoxicity and anticancer activity

The cytotoxicity assay was conducted following the 3-(4,5-dimethylthiazol-2-yl)-2,5-diphenyletetrazolium bromide (MTT) protocol described by Van de Loosdrecht et al. [14]. The breast cancerous cell line (MCF-7), hepatocellular carcinoma cell line (Hep-G2) and normal human diploid (WI-38) cell lines, obtained from the American Type Culture Collection (ATCC), was used for assessment of EA extract of AS cytotoxic or anticancer effects, respectively. Also, Taxol as positive control was assessed as anticancer agent toward both MCF-7 and HeG2 cancerous cell line. The 96-well tissue culture plate was inoculated with 1 x 10^5^ cells/ml (100 μl/well) and incubated at 37°C for 24 hours to develop a complete monolayer sheet. The growth medium was then decanted from the 96-well microtiter plates after a confluent sheet of cells was formed. The cell monolayer was washed twice with wash media. Two-fold dilutions of the tested sample were made in RPMI medium with 2% serum (maintenance medium). 0.1 mL of each dilution was tested in different wells, leaving 3 wells as controls, receiving only maintenance medium. The plate was incubated at 37°C and examined. Cells were checked for any physical signs of toxicity, e.g. partial or complete loss of the monolayer, rounding, shrinkage, or cell granulation. MTT solution was prepared (5mg/ml in PBS) (BIO BASIC CANADA INC). 20 μL of MTT solution were added to each well. The plate was placed on a shaking table, 150 rpm for 5 minutes, to thoroughly mix the MTT into the media. The plate was then incubated (37°C, 5% CO_2_) for 4 h to allow the MTT to be metabolized. The media was dumped off and the plate was dried on paper towels to remove residue if necessary. The formazan (MTT metabolic product) was resuspended in 200 uL DMSO. The plate was placed on a shaking table, 150 rpm for 5 min, to thoroughly mix the formazan into the solvent. The optical density was read at 560 nm and the background was subtracted at 620nm. The optical density should be directly correlated with cell quantity. The cell viability and inhibition percentages were calculated according to Eqs (1) and (2):

$$\text{Viability}\;\%=\;\frac{\text{Test}\;\text{OD}}{\text{Control}\;\text{OD}}\;\text{X}\;100$$ (1)

Inhibition%=100−Viability% (2)

### Antioxidant activity

EA-AS was tested for its antioxidant activity following the 2,2-diphenyl-1-picrylhydrazyl (DPPH) assay. The ability of the crude extract, at a concentration of 1000, 500, 250, 125, 62.5, 31.25, 15.62, 7.81 and 3.9 μg/mL to scavenge the DPPH radicals was examined. DPPH scavenging activity of different concentrations of EA extract of AS was determined via calculation of the percentage of antioxidant activity, in correlation to ascorbic acid (AA), following Eq (3):

$$\text{Antioxidant}\;\text{activity}\;\%=\frac{\text{Abs}.\;\text{of}\;\text{control}-\text{Abs}.\;\text{of}\;\text{sample}}{\text{Abs}.\;\text{of}\;\text{control}}\;\text{X}\;100$$ (3)

### Total phenolics content (TPC)

TPC was measured using the Folin-Ciocalteu (FC) method, as described by [15]. Specifically: 50 μL of EA-AS was pipetted into test tubes and adjusted to 1 mL by D water. 0.5 mL of 1 N FC reagent was added to each tube, including the blank, and allowed to stand for 5 minutes at 25°C. Then, 2.5 mL of 5% Na_2_CO_3_ sol was added. After 25°C for 40 minutes absorbance was measured at 725 nm. Total phenolic content was expressed as micrograms of gallic acid equivalent.

### Total flavonoid content (TFC)

TFC of EA-AS was determined using the aluminum chloride (AlCl_3_) colorimetric method: 0.5 mL of the Launaea nudicaulis extract was pipetted into a series of test tubes. The volume in each tube was adjusted to 1 mL with DH_2_O. 150 μL of 5% NaNO_2_ solution was added to all the tubes and allowed to incubate for 5 minutes at 25°C. Then, 150 μL of 10% AlCl_3_ solution was added to all the test tubes and incubated for 6 minutes at 25°C. After this, 2 mL of 4% NaOH solution was added to all the tubes, and the final volume was made up to 5 mL with DH_2_O. The test tubes were vortexed and allowed to stand for 15 minutes and absorbance was measured at 510 nm [16]. The flavonoids content of extracts was estimated by using the quercetin standard calibration curve.

### Tannin content (TC)

TC of the EA-AS was determined using the FD method, as described by Makkar [15]. Additionally, the non-tannin phenolics (NTP) were isolated and quantified: To isolate NTP, 0.5 mL of the plant extract and 0.5 mL of DH_2_O were mixed with 0.1 g of PVPP in a test tube. The mixture was incubated at 4°C for 4 hours and then centrifuged for 10 minutes. The supernatant, containing the NTP, was collected. For tannin determination, 0.5 mL of the FD reagent (1 N) and 100 μL of the NTP extract were combined, and the volume was adjusted to 1 mL with DH_2_O for each sample, including the blank. The samples were allowed to stand at 25°C for 5 minutes. Then, 2.5 mL of 5% Na_2_CO_3_ solution and incubated at 25°C for 40 minutes in the dark and measured at 725 nm. The tannin contents were determined by using Tannic as a reference compound.

### Total alkaloids content (TAC)

TAC of the EA-AS was measured quantitatively using the method described by Harborne [17]. A 1 g sample of the AS powder was mixed with a 4:1 ratio of 70% ethanol and glacial acetic acid. The mixture was left to stand for at least 6 hours and then filtered. TAC in the supernatant were precipitated by the drop wise addition of concentrated ammonia solution. TAC were then filtered and dried in an oven at 70°C until they reached a constant weight. The alkaloid content was calculated and expressed as milligrams per 100 grams of dry weight of the AS plant sample. Atropine standard solution were used in determined the alkaloids.

### GC mass analysis

The extracted compounds from AS were first dissolved in methanol (CH_3_OH). Then, the solution was dried using anhydrous sodium sulfate (Na_2_SO_4_) to remove any remaining water. Finally, the sample was passed through a 0.45 μm syringe filter before being injected into the GC-MS system. The GC-MS instrument used was a Trace GC Ultra-ISQ system from Thermo Scientific, USA. The initial column temperature was set at 70°C, then increased to 280°C at a rate of 5°C per minute and held for 2 minutes. This was followed by a further increase to 300°C at a rate of 10°C per minute. The extracted components were identified and quantified by comparing their mass spectra and retention times to the databases of known compounds from the Wiley 09 and NIST 11 libraries [18].

### Statistical analysis

The experiment was conducted in triplicate, with the reported values representing the averages of three independent trials. The data was analyzed using a one-way analysis of variance (ANOVA) model to determine if there were significant differences between the groups (p<0.05).

## 3. Results and discussion

### Antimicrobial activity

**Fig 1:**
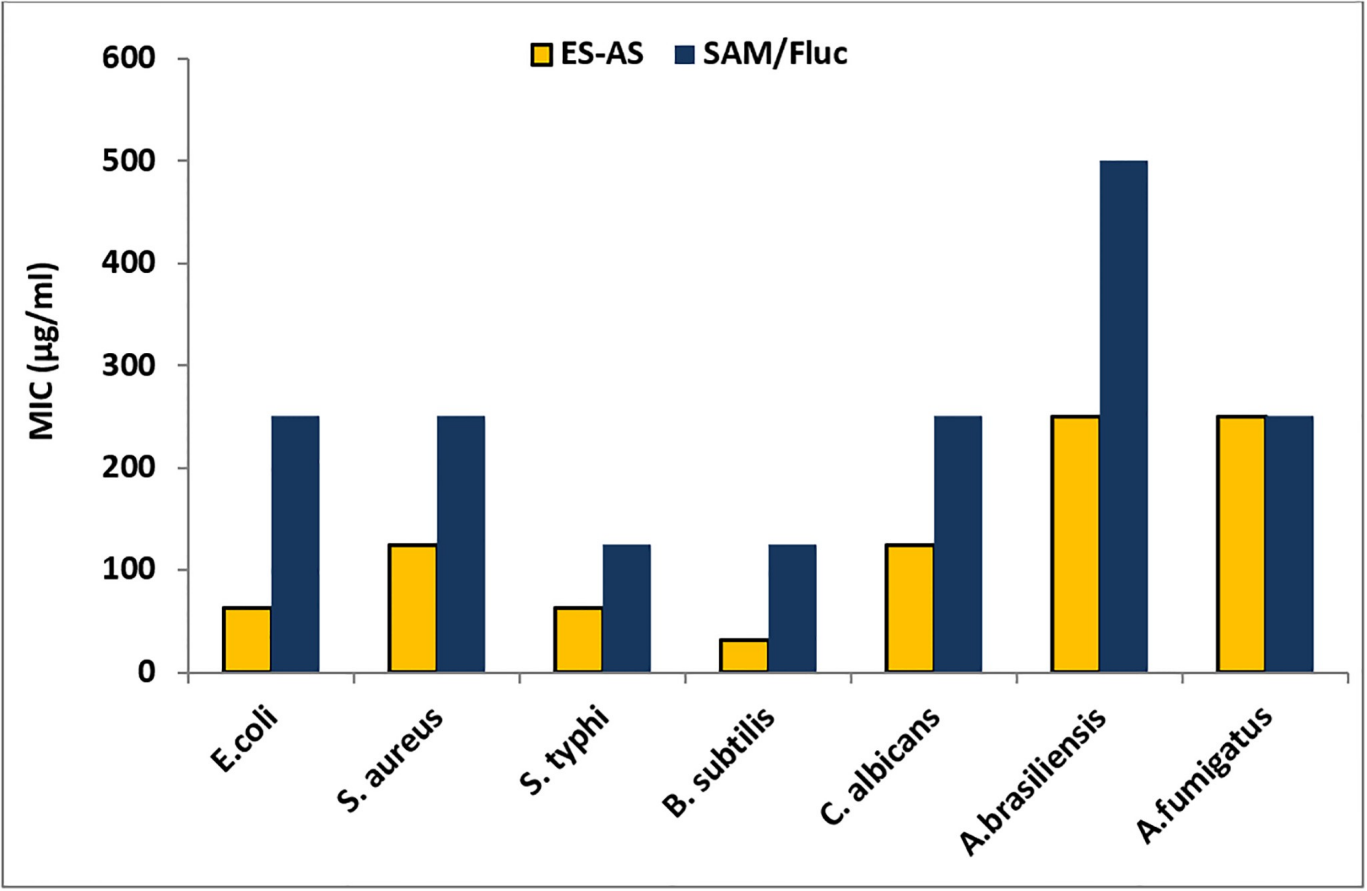
Minimum inhibitory concentrations of EA-AS and SAM/Fluc toward selected bacterial and fungal strains. * EA-AS = Ethyl acetate extract of *Anabasis setifera* leaves, Fluc = Fluconazole, SAM = Ampicillin/sulbactam, and MIC = minimum inhibitory concentration. *E*. *coli = Escherichia coli*, *S*. *aureus = Staphylococcus aureus*, *S*. *typhimurium = Salmonella typhimurium*, *B*. *subtilis = Bacillus subtilis*. *C*. *albicans = Candida albicans*, *A brasiliensis = Aspergillus brasiliensis*, *A*. *fumigatus = Aspergillus fumigatus*. Standards were used Ampicillin/sulbactam in bacteria (*E*. *coli*, *S*. *aureus*, *S*. *typhimurium* and *B*. *subtilis*) and Fluconazole in fungi (*Candida albicans*, *Aspergillus brasiliensis*, *Aspergillus fumigatus*).

According to recent studies, plant leaf extracts have shown promising antimicrobial activity [19,20]. Researchers have identified various phytochemicals, such as phenolic compounds, flavonoids, and terpenoids, within these extracts that exhibit inhibitory effects against a wide range of microorganisms, including bacteria, fungi, and viruses [21,22]. These natural antimicrobials offer a sustainable alternative to synthetic antibiotics, and their potential applications range from food preservation to the development of novel therapeutic agents. In the current study, ethyl acetate extract of *Anabasis setifera* leaves (EA-AS) was evaluated for antimicrobial activity as illustrated in Fig 1 and S1 Table in S1 File.

EA-AS exhibited outstanding antimicrobial activity toward *E*. *coli*, *S*. *aureus*, *S*. *typhimurium* and *B*. *subtilis* with inhibition zones 26.6±1.53, 25.2±1.21, 28.9±1.35 and 30.0±1.0 respectively (Table 1). Furthermore, minimum inhibitory concentrations of EA-AS against all tested bacteria were determined (Fig 1). Results illustrated that, MICs of EA-AS toward *E*. *coli*, *S*. *aureus*, *S*. *typhimurium* and *B*. *subtilis* were 62.5, 125, 62.5 and 31.25 μg/mL respectively. Ampicillin/sulbactam as a standard antibiotic showed antibacterial activity where inhibition zones were 19.16 ± 1.26, 16.0 ± 1.0, 24.83 ± 1.25 and 21.16 ± 0.76 mm toward *E*.*coli*, *S*. *aureus*, *S*. *typhimurium* and *B*. *subtilis*. Also, MICs of SAM were 250, 250, 125 and 125 μg/ml respectively. Additionally, EA-AS showed antifungal activity against *C*. *albicans*, *A*. *brasiliensis* and *A*. *fumigatus* with inhibition zones 24.33 ± 0.57, 15.96 ± 0.95 and 14.66±0.57 mm. Moreover, MIC of EA-AS extract against *C*. *albicans* was 125 μg/ml, while as MIC of EA-AS toward *A*. *brasiliensis* and *A*. *fumigatus* was 250 for both. Moreover, Fluconazole exhibited antifungal activity against *C*. *albicans*, *A*. *brasiliensis* and *A*.*fumigatus* with inhibition zones 22.27 ± 1.41, 12.0±0.9 and 16.17 ± 1.76 mm. Furthermore, MICs of Fluconazole toward *C*. *albicans*, *A*. *brasiliensis* and *A*.*fumigatus* 250, 500 and 250 μg/ml respectively.

**Table 1:** Antimicrobial activity of EA-AS.

Microbial strain	EA	EA-AS*	SAM/Fluc
*E*.*coli*	9.33±0.57^a^	26.6±1.53^bc^	19.16±1.26^bc^
*S*. *aureus*	0.00^b^	25.2±1.21^c^	16.0±1.0^c^
*S*. *typhimurium*	9.66±0.57^a^	28.9±1.35^ab^	24.83±1.25^a^
*B*. *subtilis*	10.3±0.57^a^	30.0±1.0^a^	21.16±0.76^b^
*C*. *albicans*	0.00^b^	24.33±0.57^c^	22.27±1.41^ab^
*A*. *brasiliensis*	9.66±0.57^a^	15.96±0.95^d^	12.0±0.9^d^
*A*. *fumigatus*	9.5±0.86^a^	14.66±0.57^d^	16.17±1.76^c^

*EA, EA-AS, SAM and Fluc mean ethyle actetate, Ethyl acetate extract of AS Ampicillin/sulbactam antibiotic and Fluconazole respectively. The letters a to d represented the power significance.

The genus Anabasis, a group of halophytic (salt-tolerant) plants native to arid regions of Central Asia and the Middle East, has demonstrated promising AMA. Maatalah et al. [23] reported that, alkaloids and saponin extracts of *Anabasis articulate* showed AMA against *E*. *coli* ATCC 25922, *S*. *aureus* ATCC 6538, *K*. *pneumonia*, *B*. *subtilis* ATCC 6633, *P*. *aeruginosa* ATCC 14028, *C*. *albicans* with MIC from 0.5 to 1 mg/ml. Likewise, alkaloids which extracted from *A*. *articulata* stems exhibited AMA toward bacterial and unicellular fungal strains [24]. The AMA of plant leaves crude extracts can be attributed to several mechanisms of action, including membrane disruption, enzyme inhibition, inhibition of virulence factors, induction of oxidative stress, and disruption of biofilm formation [25,26]. The phytochemicals present in our extract, such as phenols, flavonois, tannins and alkaloids, can interact with and disrupt the integrity of the microbial cell membrane, leading to the leakage of cellular contents. These bioactive compounds can also interfere with the activity of essential enzymes involved in microbial metabolism, inhibit the production or activity of virulence factors, generate reactive oxygen species, and disrupt the formation of microbial biofilms [27,28].

### Cytotoxicity and anticancer activity

**Fig 2:**
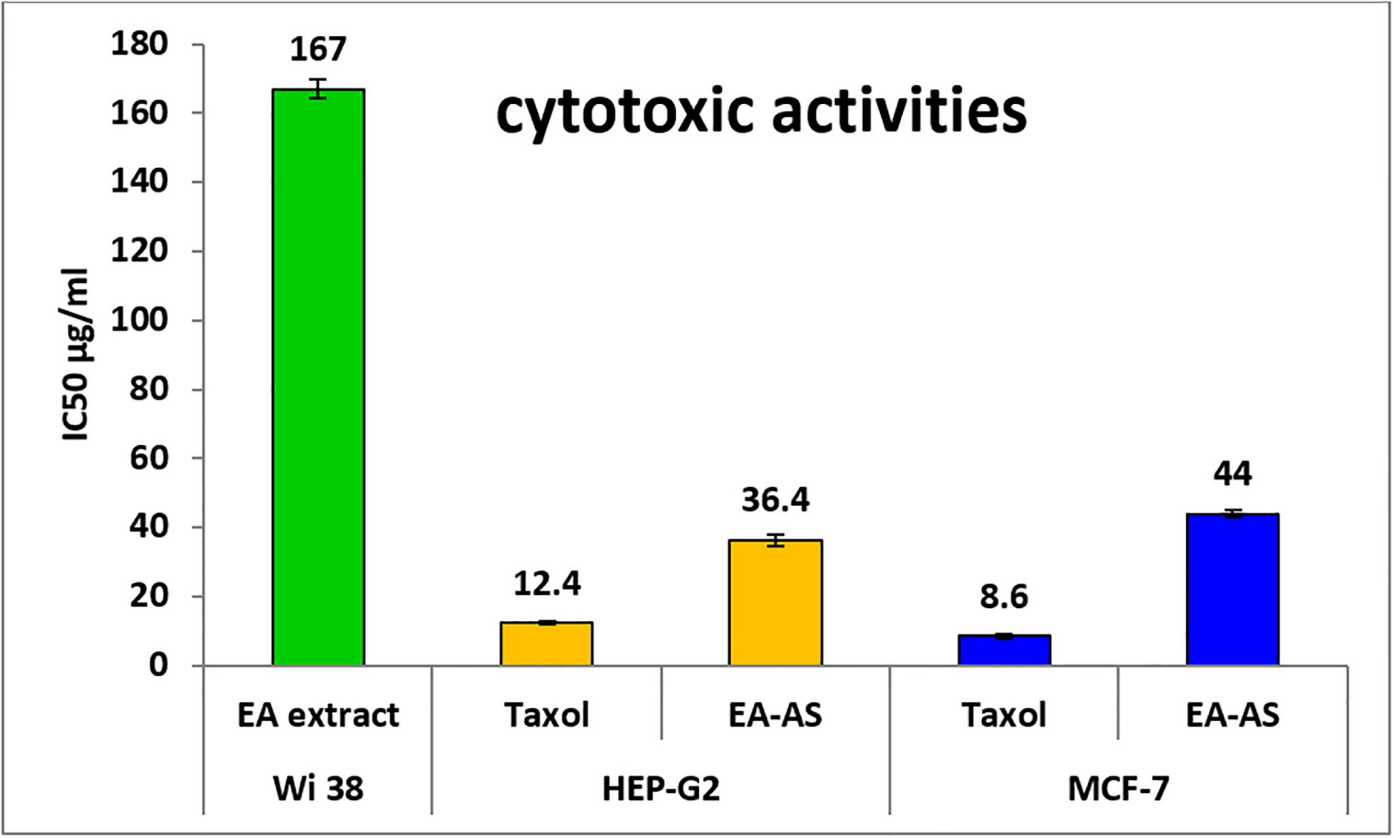
In vitro cytotoxic activities of EA-AS extract against MCF-7 and HepG2 cell lines. Data are represented as mean ± SD of three independent experiments. * EA-AS = Ethyl acetate extract of *Anabasis setifera* leaves, The breast cancerous cell line (MCF-7), hepatocellular carcinoma cell line (Hep-G2) and normal human diploid (WI-38) cell lines.

Assessing the biosafety of plant extracts using cytotoxicity toward normal cell lines is a crucial step in evaluating their safety profile through determination safe concentrations which can be used in anticancer activity [29]. This information can then be used to establish a safety margin and guide further development and testing of the extract. In the current study, Wi 38 normal cell line was selected to determine the safety of the extract. Results illustrated that, IC_50_ of EA-AS toward Wi 38 normal cell line was 167 μg/mLas shown in Fig 2. In general, if the IC_50_ is ≥ 90 μg/mL, the material is classified as non-cytotoxic [30]. Therefore, EA-AS is considered safe to use. Thus, safe concentrations and maximum non-toxic concentrations of this extract were checked for anticancer activity.

Investigating the anticancer activity of plant extracts is an area of active research and great interest in the field of natural product drug discovery. In general, plant-derived compounds have shown promising potential as anticancer agents due to their diverse phytochemical profiles, which often include bioactive secondary metabolites with cytotoxic, anti-proliferative, and pro-apoptotic properties. In this study, EA-AS was evaluated for anticancer activity against Hep-G2 and MCF-7 cancerous cell lines (Fig 2). Results revealed that, EA-AS has promising anticancer activity toward Hep-G2 and MCF-7 where IC_50_ was 36.4 and 44 μg/ml respectively. In compared to Taxol, IC_50_ of taxol was 12.4 and 8.6 μg/mL respectively. Several studies have reported that extracts from various Anabasis species exhibited cytotoxicity against a range of cancer cell lines, including those derived from breast, lung, prostate, and leukemia [31–33].

The anticancer mechanisms of plant extracts can be multifaceted, involving the modulation of key signaling pathways, the induction of cell cycle arrest, the triggering of programmed cell death (apoptosis), the inhibition of angiogenesis, and the suppression of metastasis [34]. Moreover, synergistic interactions between the diverse phytochemicals present in plant extracts can lead to enhanced anticancer activity compared to individual compounds [35]. The anticancer properties of EA-AS have been linked to several distinct mechanisms of action. Previous studies have demonstrated that Anabasis extracts, which are rich in BAC like alkaloids, flavonoids, and terpenoids, can induce apoptosis (programmed cell death) in a variety of cancer cell lines [31,33]. These EA-AS have been observed to activate caspase enzymes, key regulators of the apoptotic pathway, leading to the fragmentation of DNA and ultimately the death of cancer cells [36]. Additionally, plant extracts have demonstrated the ability to inhibit cell proliferation by disrupting the cell cycle and arresting cancer cells in specific phases, thereby preventing their uncontrolled division [37]. Furthermore, the extracts have been found to modulate cellular signaling pathways involved in cancer progression, such as the PI3K/Akt and MAPK pathways, effectively suppressing tumor growth and metastasis in in vivo animal models [34,38]. These multifaceted anticancer mechanisms highlight the therapeutic potential of Anabasis plant-derived compounds and warrant further investigation for the development of novel cancer treatments.

### Antioxidant activity

The assessment of antioxidant activity is a critical component in the comprehensive evaluation of plant extracts and their potential therapeutic applications [39]. Many plant-derived compounds, such as polyphenols, carotenoids, and terpenoids, possess potent free radical-scavenging and reactive oxygen species (ROS)-neutralizing capabilities, which can have significant implications for their use in the prevention and management of various diseases [40,41]. In this study, EA-AS was assessed for antioxidant activity as shown in Table 2 and S2 Fig in S1 File. Results revealed that EA extract of AS showed antioxidant activity where EC_50_ (concentration required to obtain a 50% antioxidant effect) was 30.6 μg/mL. In compared to positive control (ascorbic acid), EA-AS had antioxidant activity less than AA were EC_50_ of AA was 5.1 μg/mL. Senhaji et al. [42] reported that, *Anabasis aretioïdes* extract has antioxidant activity with EC_50_ = 52.91 μg/ml. Also, *Anabasis articulata* (Forssk.) Moq crude extract exhibited antioxidant activity with EC_50_ with 90 μg/mL[43]. Abdulsahib et al. [44] evaluated *Anabasis articulata* Stem Extract for antioxidant activity, where results showed potent activity with EC_50_ 94.7 μg/mL. Furthermore, leaves of *Anabasis Articulata* from Algerian habitat showed antioxidant activity using DPPH with EC_50_ ranged from 3.200 ± 0.088 to 4.900 ± 0.130 μg/mL [45]. The antioxidant activity of the extract is associated with the presence of compounds with high antioxidant activity, as proven by GC mass analysis. The GC mass analysis of the extract confirmed the presence of the following compounds: hexa-2,4-diyn-1-ylbenzene, nerolidol, Spathulenol, 2-naphthalenemethanol, decahydro-4-trimethyl-8-methylene, hexadecenoic acid, tremetone, desmethoxyencecalin, 13-heptadecyn-1-ol, dotriacontane, taylorione, retinoic acid, benz[e]acephenanthrylen-3a-(1h)-ol, 2,3-dihydro, methyl commate d, and pectolinaringenin.

**Table 2:** Antioxidant activity of EA-AS using DPPH method.

Conc. (μg/mL)	Ascorbic acid		EA-AS	
	Antioxidant Activity %	EC_50_ (μg/mL)	Antioxidant Activity %	EC_50_ (μg/mL)
**2000**	99.07±0.12^a^	5.1	98.67±1.15^a^	30.6
**1000**	98.67±0.58^a^		98.33±0.58^a^	
**500**	98.00±1.00^a^		93.83±1.04^b^	
**250**	94.13±0.81^b^		89.17±0.97^c^	
**125**	88.73±1.42^c^		81.47±1.36^d^	
**62.5**	76.67±0.58^d^		69.13±1.03^e^	
**31.25**	70.83±0.76^e^		52.90±1.01^f^	
**15.625**	64.33±1.53^f^		38.67±1.53^g^	
**7.81**	55.93±0.90^g^		27.80±1.31^h^	
**3.9**	47.00±1.00^h^		10.17±1.04^i^	

Letters a,b,c,…. Mean significance power.

### Phytochemical analysis

**Fig 3:**
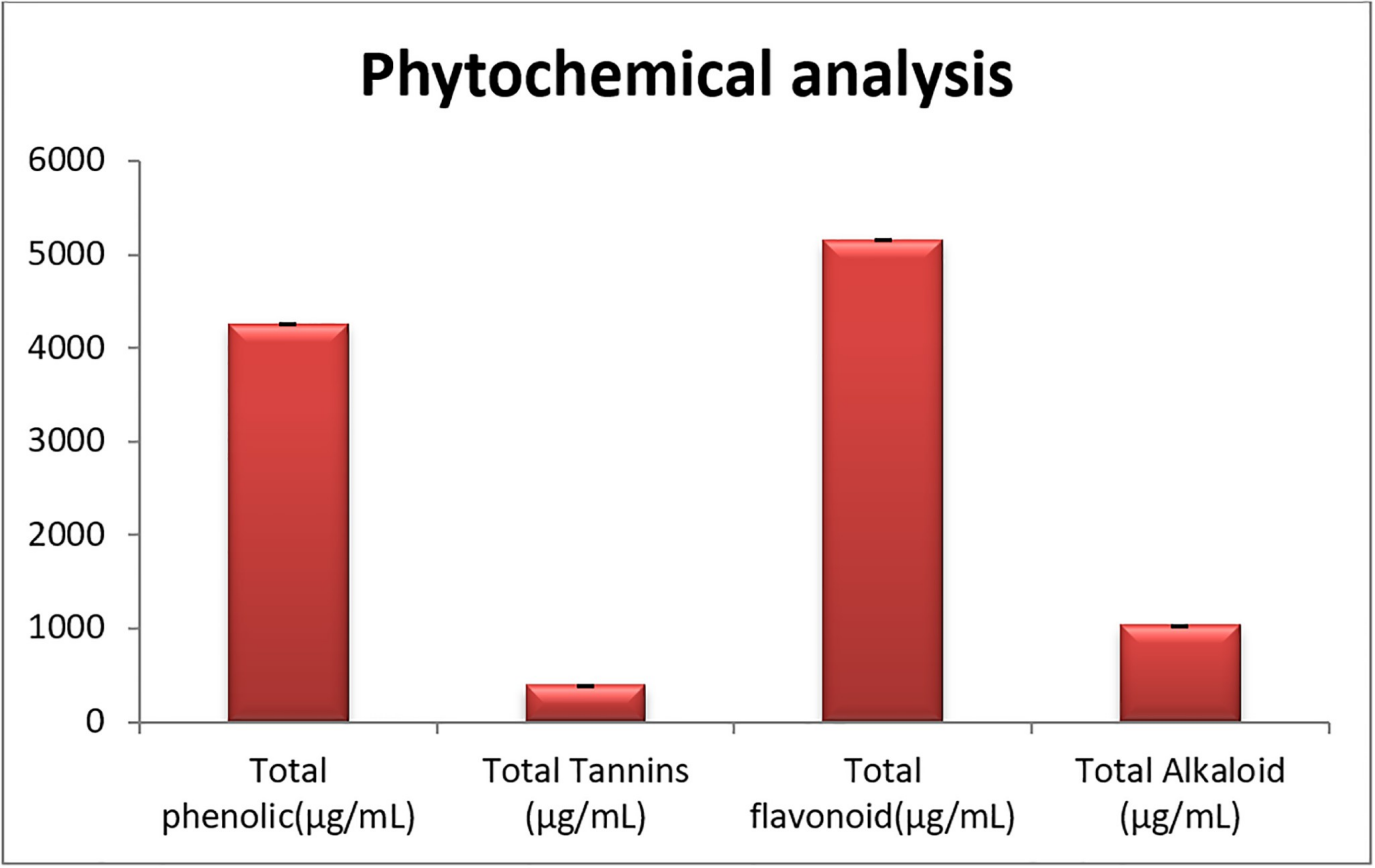
Quantitative phytochemical analysis of *Anabasis setifera*.

The result of the total phenolic content of AS being 4,264 μg /ml is a promising result and suggests that the plant may have significant biological and pharmacological potential (Fig 3) and S2 Table in S1 File. The AS may have significant antioxidant, anti-inflammatory, and AMA. The results of this study corroborate the findings reported by Gheraissa et al. [46] which indicated that AS exhibits antibacterial activity specifically against *S*. *aureus*, *L*. *innocua*, and *E*. *coli*. Tannins are a class of polyphenolic compounds that have been linked to several beneficial activities, including antioxidant, anti-carcinogenic, and AMA [47]. The total tannin content of the AS extract was found to be 391.17μg/ml. The presence of tannins in the AS extract suggests that it may have potential applications in various industries, including nutraceuticals, pharmaceuticals, and cosmetics. Flavonoids are a diverse group of plant-derived compounds that are renowned for their antioxidant, anti-inflammatory, and neuroprotective properties [48]. The total flavonoid content of the AS sample was found to be exceptionally high, reaching 5,163 μg/mL (Fig 3). This remarkably elevated flavonoid concentration further reinforces the potential bioactive and therapeutic capabilities of this plant extract. The abundance of these beneficial flavonoids suggests that AS may possess a wide range of valuable medicinal applications. The total alkaloid content of the *EA-AS* was found to be 1036.26 μg/ml. Alkaloids are a class of nitrogen-containing organic compounds that are known for their pharmacological activities. Phenolics, tannins, flavonoids, and alkaloids have significant important for maintaining health and preventing disease as well as nutritional supplements. Thus, they play significant roles in traditional and modern medicine, showcasing their potential for various therapeutic applications, including antioxidant, anti-inflammatory, and antimicrobial properties.

### GC-MS

**Fig 4:**
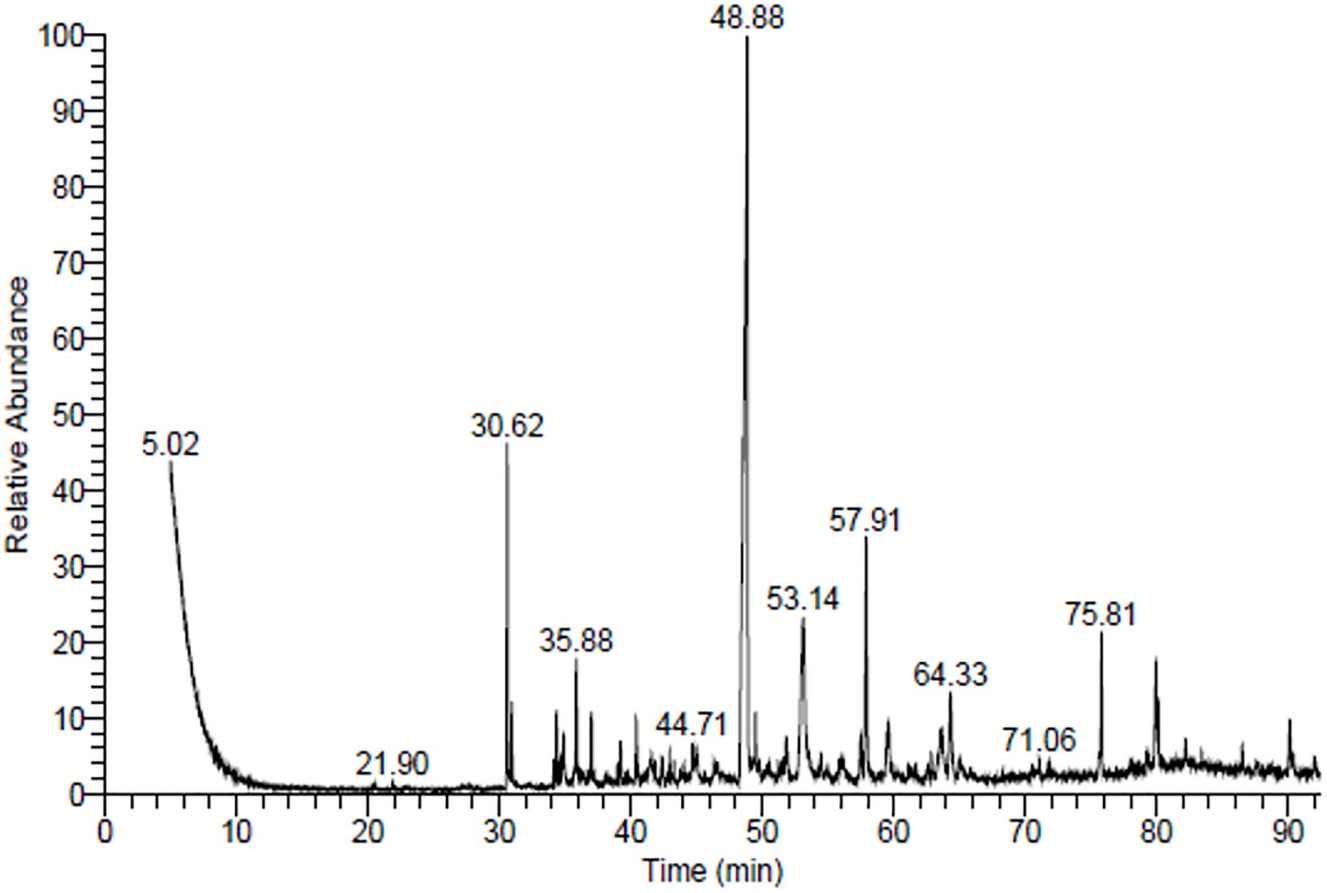
Gas chromatography mass spectrophotometry (GCMS)of Ethyl acetate extract of *Anabasis setifera* leaves.

EA-AS plant was found to contain 55 significant compounds that were identified through GC mass analysis. The most major compounds were 1,4-dimethoxy-6,7,8 9-tetrahydro-5-benzocycloheptenone (26.04%), hexa-2,4-diyn-1-ylbenzene (8.40%), dihydrobe nzo [b] fluoranthene (6.10%), ethanone, 1-[2,3-dihydro-2-(1- methylethenyl)-5-benzofuranyl (6.10%), and valerenol (4.08%) (Table 3 in S1 File and Fig 4). 1,4-dimethoxy-6,7,8,9-tetrahydro-5-benzocycloheptenone is a derivative of benzocycloheptenone with two methoxy substituents. It has been found to possess multiple biological activities, including antioxidant, anti-inflammatory, hepatoprotective, neuroprotective, and antiviral properties (46). Hexa-2,4-diyn-1-ylbenzene is an aromatic organic compound identified in the extract of plants and it has been found to exhibit anticancer, antioxidant, and antifungal activities [47]. Ethanone, 1-[2,3-dihydro-2-(1-methylethenyl)-5-benzofuranyl], is a chemical compound with the molecular formula C14H14O. It is a derivative of benzofuran, a heterocyclic compound with a six-membered ring containing an oxygen atom. It serves as a precursor to various pharmaceuticals and has been used in the synthesis of several compounds with biological activities [48]. Valerenol is a bicyclic sesquiterpenoid alcohol found in plants, known for its anxiolytic (anti-anxiety) activity, making it a compound of interest for potential therapeutic applications in mental health and nervous system disorders [49].

**Table 3:** GC–MS of AS.

No.	Compound name	RT (min)	Peak area %	Activity	References
1	Hexa-2,4-diyn-1-ylbenzene	30.62	8.40	Anticancer, antioxidant, antifungal activity	[50]
2	Benzene, 1-(1,5-dimethyl-4-hexenyl)-4-methyl	30.97	1.39	Antifungal, and antibacterial Activity	[51]
3	Acorenol	32.23	0.80	Antibacterial Activity	[52]
4	Nerolidol	34.15	0.48	Antioxidant,AMA, ant biofilm, immunomodulatory, antimalarial, and anti-inflammatory	[53,54]
5	Spathulenol	34.37	1.47	Antioxidant, antifungal, anti-inflammatory, and anti-hyperalgesic	[55]
6	Citronellyl valerate	34.64	0.36	Mutagenic activity	[56]
7	2-Fluorobenzoic acid. heptadecyl ester	34.97	1.05	AMA	[57]
8	Cis-isoapiole	35.70	0.12	Vasorelaxation, AMA, and antimalarial activities	[58]
9	Hexadecane	36.18	0.18	AMA	[59]
10	2-Naphthalenem ethanol, Decahydro-4-trimethyl-8- methylene	37.02	1.44	AMA, and antioxidant activity	[60]
11	Hexadecenoic acid	38.97	0.30	Antioxidant, anti-inflammatory, AMA, and anti-cancer activities	[61]
12	Tremetone	39.22	1.00	Analgesic, and antioxidant activity	[62]
13	Desmethoxyencecalin	40.44	1.45	Anti-inflammatory, antioxidant, anti-cancer	[63]
14	7-epi-cis-sesquisabin ene hydrate	41.59	0.59	AMA and anti-inflammatory	[64]
15	13-Heptadecyn-1-ol	41.88	0.37	AMA, and antioxidant activity	[65]
14	Thunbergol	42.41	0.89	Antioxidant activities	[66]
15	1-Hexadecanol	42.96	0.54	AMA, antioxidant, and cytotoxicity and anticancer Activity	[67,68]
16	Gitoxigenin	43.16	0.22	Antiviral, and anticancer activity	[69]
17	Dotriacontane	43.30	0.41	AMA, antioxidant, and anticonvulsant activity	[70,71]
18	Taylorione	44.71	1.56	AMA, antioxidant, and cardiotonic activity	[72,73]
19	Furanether	46.62	0.34	Anticancer activity	[74]
20	Ethylestrenol	46.94	0.17	Anabolic activity	[75]
21	Ligulatin	47.76	0.22	AMA, and antioxidant	[76]
22	Valerenol	48.42	4.08	Anxiolytic activity	[77]
23	1,4-Dimethoxy-6,7,8 9-tetrahydro-5-benzo cycloheptenone	48.88	26.04	Antioxidant, anti-Inflammatory, Hepatoprotective, neuroprotective, antiviral activity	[78,79]
24	Retinoic acid	49.18	0.22	Antioxidant, and anti-inflammatory	[80,81]
25	Falcarinol	49.49	1.04	Anticancer, antioxidant, and AMA	[82]
26	Isochiapin B	49.78	0.90	AMA, and antioxidant	[83]
27	Terretonin	50.55	0.27	AMA	[84]
28	Epipallensin	51.23	0.44	AMA	[85]
29	Powelline	51.55	0.43	AMA	[86]
30	Ethanone, 1-[2,3-dihydro-2-(1- methylethenyl)-5-ben zofuranyl]	53.05	6.10	AMA, and antifungal activity	[48]
31	Isocalamenediol	53.55	0.21	Cardiac protective	[87]
32	Octadecanoic acid	54.48	0.47	Cardiovascular Health, anti-inflammatory activity.	[88]
33	Digoxigenin	55.02	0.57	Anticancer activity	[89]
34	Tibolone	55.57	0.14	Anticancer activity	[90]
35	Docosanol	55.85	0.26	Antiviral activity	[91]
36	Artemisetin	56.56	0.11	Antimalarial, anticancer, anti-inflammatory, antioxidant, and neuroprotective activity	[92]
37	Benz[e]acephen anthrylen-3a- (1h)-ol, 2,3-dihydro	57.48	1.21	Antibacterial, antifungal, anti-inflammatory, and antioxidant activity	[93]
38	Dihydrobe nzo[b]fluoran thene	57.91	6.10	Mutagenic	[94]
39	Methylprednisolone	58.84	0.14	Anti-inflammatory, and antitumor activity	[95]
40	Methyl-à-cyano-á-methoxy-p-nit rocinnamate	59.58	2.79	AMA	[96]
42	Pregnan-20-one	61.10	0.32	Neuroprotective	[97]
42	Androst-2-en-4-one, 17-hydroxy	61.35	0.28	Neuroprotective, and AMA	[98]
43	Phenol, 2,2’-methylenebis Dimethylethyl methyl	61.69	0.35	Antioxidant activity	[99]
44	Hahnfett	62.60	2.11	Antibacterial	[100]
45	Benzo, indene hexahy dro-7-methoxy-3-oxo dimethyl	63.60	1.21	Antibacterial, and fungicidal	[66]
46	Phenanthrene, ctahydro-trim ethyl-7-(methylethyl)	64.32	2.33	AMA	[101]
47	Hexadecanoic acid,	64.95	0.20	Antioxidant and AMA	[61]
48	Astaxanthin	71.70	0.14	Antioxidant, and neuroprotective	[102]
49	Pectolinaringenin	75.63	0.25	Antioxidant, and neuroprotective	[103]
50	Nonacosane	75.80	2.44	Nematicidal	[104]
51	Dimethyl 2,3-bis(phenylethynyl)fumarate	79.72	2.38	Antifungal activity	[105]
52	Dotriacontane	80.13	0.90	AMA, antioxidant, and antiviral	[70]
53	Stigmasta-5,20(22)-dien-3-ol	82.22	0.87	Antioxidant, anti-inflammatory, cholesterol-lowering, cardioprotective and Anti-cancer	[106]
54	Sitosterol	83.43	0.30	Anticancer activity	[107]
55	Methyl commate d	86.50	0.86	Antioxidant and antimutagenic activities	[108]

* AMA (antimicrobial activity).

The antioxidant properties of AS have been studied, and the GC mass analysis confirmed this by detecting many compounds with antioxidant activity, including hexa-2,4-diyn-1-ylbenzene, nerolidol, Spathulenol, -naphthalenem ethanol, decahydro-4-trimethyl-8-methylene, hexadecenoic acid, tremetone, desmethoxyencecalin, heptadecyn-1-ol, thunbergol, hexadecanol, dotriacontane, taylorione, ligulatin, retinoic acid, and falcarinol. Furthermore, the antioxidant properties of *Anabasis setifera* have been linked to a range of biological activities, including anti-inflammatory, AMA and anticancer effects. There were many compounds showed by GC mass have antimicrobial activity including, benzene, 1-(1,5-dimethyl-4-hexenyl)-4-methyl, acorenol, 2-fluorobenzoic acid. heptadecyl ester, cis-isoapiole, hexadecane, 2-naphthalenem ethanol, decahydro-4-trimethyl-8- methylene, hexadecenoic acid, 7-epi-cis-sesquisabin ene hydrate, 13-heptadecyn-1-ol, 1-hexadecanol, dotriacontane, taylorione, ligulatin, falcarinol, isochiapin B, terretonin, epipallensin, eowelline, ethanone, 1-[2,3-dihydro-2-(1- methylethenyl)-5-ben zofuranyl], Digoxigenin, tibolone, docosanol, artemisetin, benz[e]acephen anthrylen-3a- (1h)-ol, 2,3-dihydro, methyl-à-cyano-á-methoxy-p-nit rocinnamate, androst-2-en-4-one, 17-hydroxy, hahnfett, Phenanthrene, ctahydro-trim ethyl-7-(methylethyl), hexadecanoic acid, dotriacontane. There were many compounds showed by GC mass have anticancer activity including, hexa-2,4-diyn-1-ylbenzene, hexadecenoic acid, desmethoxyencecalin, 1-hexadecanol, gitoxigenin, furanether, falcarinol, digoxigenin, tibolone, docosanol, artemisetin, and sitosterol.

## 4. Conclusion

The comprehensive analysis of the *Anabasis setifera* leaf extract (EA-AS) reveals its immense potential as a rich source of valuable phytochemicals with diverse bioactivities. The extract exhibited potent AMA against a range of pathogenic microbes, suggesting its possible applications in AMA. Additionally, the extract demonstrated significant anticancer properties against liver and breast cancer cell lines, underscoring its potential as a natural anticancer agent. The GC-MS analysis of EA-AS identified a plethora of antioxidant compounds, corroborating the extract’s impressive antioxidant capacity. The exceptionally high contents of phenols, tannins, flavonoids, and alkaloids further highlight the phytochemical wealth of this plant, indicating its vast applications in the nutraceutical, pharmaceutical, and cosmetic industries. These findings collectively suggest that AS leaf extract is a treasure trove of bioactive phytochemicals with multifaceted therapeutic and industrial potential. Further in-depth studies on the mechanistic aspects and in vivo efficacy of this extract are warranted to unlock its full pharmaceutical and commercial prospects. The study requires further research to be beneficial, such as applying this extract in vivo, comparing it with currently used materials, and mixing them. Additionally, the extract should be applied on a more comprehensive scale.

## Supporting information

S1 File Supplementary file containing Table S1: Minimum inhibitory concentrations of EA-AS toward selected bacterial and fungal strains, Table S2: Phytochemical analysis, and Fig S1: Antioxidant activity of EA-AS using DPPH method. (DOCX)
