# Supplementary material for: Anabasis setifera leaf extract from arid habitat: A treasure trove of bioactive phytochemicals with potent antimicrobial, anticancer, and antioxidant properties
Source: PLoS One. 2024 Oct 25;19(10):e0310298. doi: 10.1371/journal.pone.0310298 (PMC11508485; doi:10.1371/journal.pone.0310298)
Supplement: S1 File — (DOCX) [file pone.0310298.s001.docx]

**Table S1:** Minimum inhibitory concentrations of EA-AS toward selected bacterial and fungal strains

| **Microbial strain** | **ES-AS** | **SAM/Fluc** |
| --- | --- | --- |
| ***E.coli*** | 62.5 | 250 |
| ***S. aureus*** | 125 | 250 |
| ***S. typhi*** | 62.5 | 125 |
| ***B. subtilis*** | 31.25 | 125 |
| ***C. albicans*** | 125 | 250 |
| ***A.brasiliensis*** | 250 | 500 |
| ***A.fumigatus*** | 250 | 250 |

**Table S2:** Phytochemical analysis

| **flavonoid con. ug(QuE)/ml** | | | **Mean** | **STD** | **SE** |
| --- | --- | --- | --- | --- | --- |
| 5163.5 | 5166.62 | 5160.20 | 5163.44 | 3.210 | 1.05 |
| **Total phenolic con. ug(gal)/ml** | | | **Mean** | **STD** | **SE** |
| 4265.9 | 4265.90 | 4260.20 | 4264.00 | 3.291 | 1.07 |
| **Total Alkaloid (ug/ml)** | | | **Mean** | **STD** | **SE** |
| 1038.59 | 1034.49 | 1035.69 | 1036.26 | 2.108 | 0.69 |
| **Total Tannins conc.mg(TanE)/gm** | | | **Mean** | **STD** | **SE** |
| 391.5 | 390.40 | 391.60 | 391.17 | 0.666 | 0.22 |

**Figure S1:** Antioxidant activity of EA-AS using DPPH method.
